# The podiatric surgery theatre environment in the UK; is it conducive to learning? A quantitative study using the surgical theatre educational environment measure (STEEM)

**DOI:** 10.1186/s13047-018-0312-z

**Published:** 2019-01-15

**Authors:** Thomas Austen, Simon Otter

**Affiliations:** 1 Podplus Ltd, The Julie Rose Stadium, Ashford, Kent TN24 9QX UK; 2 School of Health Sciences, University of Brighton, Robert Dodd, Darley Road, Eastbourne, BN20 7UR UK; 3 School of Health Sciences, University of Brighton, Robert Dodd, Darley Road, Eastbourne, BN20 7UR UK

**Keywords:** Podiatric surgery, Surgical theatre educational environment, Podiatric surgery teaching and learning

## Abstract

**Background:**

In 2015 the Health and Care Professions Council (HCPC) reported that annotation of the register for podiatric surgery would improve the way in which risks are currently managed. The academic institutions provide the teaching environment for the ‘learnt’ Diploma in principles of podiatric surgery however the podiatric surgery departments facilitate the production of the next generation of podiatric surgeons. This research aimed to identify the major elements that contribute to the educational environment, and find and utilise a valid assessment tool which could identify discrete areas to be targeted for improvement as well as being used for monitoring of the environment.

**Methods:**

A quantitative study using the Surgical Theatre Educational Environment Measure (STEEM) via an online tool was utilised for podiatrists working within podiatric surgery, podiatric surgical trainees and podiatric surgeons working towards the Certificate of Completion of Podiatric Surgery Training (CCPST) with a view to assessing the educational environment within the podiatric surgical theatre in the UK.

**Results:**

16/33 responses with a response rate of 48.4% the overall STEEM mean score was 122/160. Four subscales included teaching and training, learning opportunities, atmosphere, and workload/supervision/support were measured. The overall mean score of 76.73% suggests the learning environment may be considered satisfactory; however, areas for potential improvement are identifiable. Results reveal strengths such as a non-discriminatory surgical theatre atmosphere on racial grounds.

**Conclusions:**

Perception was of a very satisfactory ‘Atmosphere’ within the theatre environment and a very satisfactory ‘opportunity to assist’ within the podiatric surgery theatre environment. The STEEM has potential to be applied further as a quality assessment tool whose results could be used to demonstrate part of the HCPC standards.

## Background

In 2015 the Health and Care Professions Council (HCPC) reported that annotation of the register for podiatric surgery would improve the way in which risks are currently managed. They published professional standards that should be met to enable annotation. The reasons given for this were that annotation would enable specific standards to be set for podiatric surgery training and practice. Training programmes would be approved and linked to the annotation, providing independent quality assurance. Annotating the Register would provide information to members of the public about who had completed recognised, approved training, supporting informed choices [1].

The learning environment considered for podiatric surgery is a workplace environment rather than a classroom environment with taught lectures. This work environment can include the outpatient clinic with new patient assessments, pre-operative assessments and post-operative reviews, dressing clinics as well as the actual theatre environment.

**Fig. 1:**
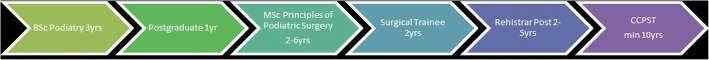
The podiatric surgery route

The podiatric surgery route (Fig. 1) does have a broad framework, and it can be considered that the consultant acts as a mentor to guide the student through this process. The whole process can be seen as formal however due to the nature of the experiential learning, the environment can be considered to be informal [2].

This unstructured format is described as informal learning which has a naturally occurring form of learning based in and around personal experience of situations [3]. This is against formal learning which has been described as having one of a number of characteristics including; having a prescribed learning framework, an organised learning event or package, the presence of a designated teacher or trainer, the award of a qualification or credit and the external specification of outcomes [4].

In their review of workplace learning, Manuti et al. [2] suggest informal learning occurs within environments where the principal objective is not learning. However learning is activated by some anticipated or actual problem which arises. They suggest informal learning could happen as a result of evolving activities such as problem solving, hypothesis testing, mentoring, coaching and job shadowing. This learning requires a mix of individual characteristics to be actioned, notably Beckett and Hager [5] report intellectual curiosity, self-directedness and self-efficacy can be key to the quality of learning within learning environments.

It has also been explained as implicit learning, deliberative learning and reactive learning. Implicit and deliberative being intentional and non-intentional with reactive learning is an ‘in between’ category, which describes situations where the learning takes place almost spontaneously, rather than planned and is in response to recent, current, or imminent situations without any time being planned or specifically set aside for it [4]. Eraut’s [6] work on informal learning in the workplace and Ajjawi and Higgs [7] provide insight into a very unstructured environment. They give insight into the factors that affect learning and how this is communicated in the clinical setting.

Ajjawi and Higgs [7] used a qualitative approach to delve deeply into the way physiotherapists communicate their clinical reasoning to patients as well as more junior physiotherapists. They took a hermeneutic phenomenological approach, essentially getting a human experience view on this subject, to gain their insight. This included observation, written reflective pieces from the subjects and interviews with subsequent analysis. It appeared the methodology was well considered and their specificity within the study of physiotherapists with a speciality may be a restrictive factor limiting its potential for other specialities.

Their findings did suggest a number of themes can affect the learning environment. They suggest the team environment, professional attributes and workplace culture all to be factors that are important. Along with this, incidents and analysis of these incidents including reflective practice appear to be important. They also found that learning to reason and to communicate reasoning are situated, embedded, and enriched in practice itself, suggesting that the learning is best done in the workplace environment.

Eraut [6] uses his previous work in 2000 [4] to consider ‘key concepts’ of informal learning which are stated as learning from experience, tacit knowledge, transfer of learning and intuitive practice to discuss the factors that he feels are effecting learning in the workplace. He highlights factors which he feels influence learning but does stress their importance will differ in different situations with different people. These factors are given as challenge and value of work, feedback, support, confidence and commitment which are given as learning factors. There are also context factors which are allocation and structuring of work, expectations and encounters/relationships with people at work. Both this theory and the findings of Ajjawi and Higgs [7] do point to the workplace environment contributing to and being a large factor in learning.

Eraut [6] interestingly does recommend that each organisation assesses their own environment to self-evaluate with a view to trying to create a positive impact on retention, innovation and quality. This is where the podiatric surgical theatre is a key environment that can be evaluated.

The teaching and learning environment utilised for podiatric surgery is both in the classroom and within the work environment including the podiatric surgery theatre. The academic institutions provide the teaching for the ‘taught’ MSc in Principles of Podiatric Surgery while the podiatric surgeons are given the trainer’ responsibilities for the clinical experience and guidance through the surgical trainee and registrar posts.

With more structured processes and within a framework which meets higher governance standards and contributes to the legislative process, under the HCPC, the evidence of quality of the institution guiding the production of new podiatric surgeons is to be monitored.

This appears to be occurring in a collaborative manner within Higher Education Institutions and the NHS to provide a framework from the start to finish of qualification for podiatric surgery. The example given by the HCPC was of NHS Education for Scotland (NES) which has recently developed a three-year, work-based podiatric surgery training programme in collaboration with Queen Margaret University, which will award a certificate of completion of training (CCT). Standards published by the HCPC require demonstration of a number of specific statements (Table 1). During the training examinations do have independent assessors who maintain the aims and objectives of surgery within criteria set by the College. However, it appears the only evidence of monitoring and evaluation that is specific to podiatric surgery is the outcomes of surgical procedures themselves. This is via the PASCOM (Podiatric and Surgical Clinical Outcome Measurement) online system which is aimed at being an audit and outcome tool [8].

**Table 1:** HCPC Statements for podiatric surgery

B. 3	The programme must have regular monitoring and evaluation systems in place
C. 3	Integration of theory and practice must be central to the curriculum
D. 3	The practice placement must provide a safe and supportive environment
D. 4	The education provider must maintain a thorough and effective system for approving and monitoring all practice placements

The operating theatre has a unique atmosphere and therefore can be seen as a specific environment in which to learn, it provides quality uninterrupted time for the teacher and learner which should be utilised for clinical teaching and learning [9]. Our primary aim was to measure the podiatric surgery theatre as an educational environment currently used by podiatrists, podiatric surgical trainees and podiatric surgery registrars for CCT in podiatric surgery. This work also seeks to identify elements that contribute to the educational environment, and find areas that may be targeted for improvement.

An inventory for measuring the learning environment in the surgical operating theatre as perceived by basic surgical trainees was created by Cassar at the University of Aberdeen in Scotland [10]. Developed on from the Postgraduate Hospital Educational Environment Measure (PHEEM), this 40-item questionnaire evolved through a literature review and input of surgical trainers and trainees via interviews. The inventory was then validated by being administered to Scottish basic surgical trainees and called the “Surgical Theatre Educational Environment Measure” (STEEM). Thus, the major elements that contribute to the educational environment in the Scottish surgical theatre were determined, and provides a valid assessment tool to identify discrete areas to be targeted for improvement.

This tool has been applied to assess teaching and learning in the surgical environment. It appears this has not been applied to the podiatric surgery theatre. In fact the author could find no literature regarding teaching and learning within the podiatric surgery theatre environment. Given the HCPC standards discussed it is possible this could be used in some part as evaluation of theatre based training.

This research aimed to identify the major elements that contribute to the educational environment, and find and utilise a valid assessment tool which could identify discrete areas to be targeted for improvement as well as being used for monitoring of the environment.

## Methods

### Materials and methods

The STEEM questionnaire consisting of 40 questions was used. Each question had a Likert scale ranging from 4 “strongly agree” to 0 “strongly disagree”. A Likert scale 0–4 has been used due to the possible overestimation of a 1–5 score [11]. Reverse coding of the negative questions; 8, 11, 14, 16, 19, 22, 23, 26, 27, 28, 30,31, 33, 34, 35, 36, 37, 38 and 40, was utilised and therefore the higher the score the more positive the perception. A maximum overall score would be of 160 and minimum score of 0 (see Table 2).

**Table 2:** shows the individual questions median score and interquartile range (IQR) with reverse coded questions marked with an asterix

No.	Statement	Median (IQR) (*N* = 16)
1	My trainer has a pleasant personality	4 (2.5–4)
2	I get on well with my trainer	4 (2.5–4)
3	My trainer is enthusiastics about teaching	3.5 (3–4)
4	My trainer has a geniune interest	4 (3–4)
5	I understand what my trainer is trying to teach me	4 (3–4)
6	My trainer’s surgical skills are very good	4 (3–4)
7	My trainer’s gives me time to practice surgical skills in theatre	4 (3–4)
8	My trainer immediately takes the instruments away when I do not perform well *	3 (2–4)
9	Before the operation my trainer discusses the surgical technique planned	3 (1–3)
10	Before the operation my trainer discusses what parts of the procedure I will perform	2.5 (1–4)
11	My trainer expects my surgical skills to be as good as his/hers *	2 (1–3)
12	My trainer gives me feedback on my performance	3.5 (3–4)
13	My trainer’s criticism is constructive	3 (2.4)
14	On this unit the type of operations are too complex for my level *	3.5 (3–4)
15	The elective operating list has the right case mix to suit my training	4 (3–4)
16	There are far too many cases on the elective list to give me opportunity to operate *	3 (2–4)
17	I get enough opportunity to assist	4 (4–4)
18	There are enough theatre sessions per week for me to gain the appropriate experience	4 (2.5–4)
19	More senior trainees take my opportunities to operate *	2.5 (2–4)
20	The number of emergency procedures is sufficient for me to gain the appropriate experience	2 (2–2.5)
21	The variety of emergency cases gives me the appropriate exposure	2 (2–3)
22	My trainer is in too much of a rush during emergency cases to let me operate	2 (2–3)
23	I miss out on operative experience because of restrictions on working hours *	3 (2.5–4)
24	I have the opportunity to develop my skills required at my stage	3 (2.5–4)
25	The atmosphere in theatre is pleasant	4 (3–4)
26	In theatre I don’t like being corrected in front of medical students, nurses and residents *	4 (3–4)
27	The nursing staff dislike it when I operate as the operation takes longer *	3.5 (2–4)
28	The anaesthetists put pressure on my trainer to operative him/herself to reduce anaesthetic time *	4 (2–4)
29	The theatre staff are friendly	4 (3–4)
30	I feel discriminated against in theatre because of my sex *	4 (3.5–4)
31	I feel discriminated against in theatre because of my race *	4 (4–4)
32	I feel part of a team in theatre	4 (3–4)
33	I am too busy doing other work to go to theatre	4 (2–4)
34	I am often too tired to get the most out of theatre teaching *	4 (2–4)
35	I am so stressed in theatre that I do not learn as much as I could *	4 (3–4)
36	I am asked to perform operations alone that I do not feel competant at *	4 (3.5–4)
37	When I am in theatre, there is nobody to cover the ward	4 (2–4)
38	I get bleeped during operations *	4 (2–4)
39	The level of supervision in theatre is adequate for my level	4 (3–4)
40	Theatre sessons are too long *	4 (3–4)

### Data collection

A link to Survey Monkey, where the STEEM had been inputted, was emailed via The College of Podiatry to podiatrists registered on a training programme and podiatrists working towards the Certificate of Completion of Podiatric Surgery Training (CCPST).

### Subjects and setting

The College of Podiatry was asked to facilitate the communication to registered trainees and registrars. This included the Deanery of Podiatric Surgery (North, Midlands and South). The inclusion and exclusion criteria were considered by the College of Podiatry, UK. This was for the attention of “podiatrists training or working within a podiatric surgery unit” as well as stating the survey cannot be completed by anyone who holds the Certificate of Completion of Podiatric Surgery Training.

As such, the database allowed the email to be sent to members who were: registered as a podiatric surgical trainee (25 email addresses) registrars (8 email addresses) which resulted in 33 eligible people on the records of the College of Podiatry.

### Data analysis

Statistics were used to report the median and interquartile ranges. Within the STEEM there are 4 subscales ‘Teaching and training’, ‘Learning Opportunities’, ‘Atmosphere’ and ‘Supervision/workload/support’ which is analysed with descriptive statistics utilising graphic representation. The highest and lowest scored statements was noted and discussed within the context of podiatric surgery. Comparisons are made against the original Cassar [10] study and the most recent Binsaleh [12] study.

## Results

Sixteen out of thirty three responded. The overall score was a total of 1952 out of a maximum 2544 with a mean score of 122/160, as a percentage this is 67.73%. Figure 1 shows the median and interquartile range for each statement in the STEEM survey for the whole group (*n* = 16).

**Fig. 2:**
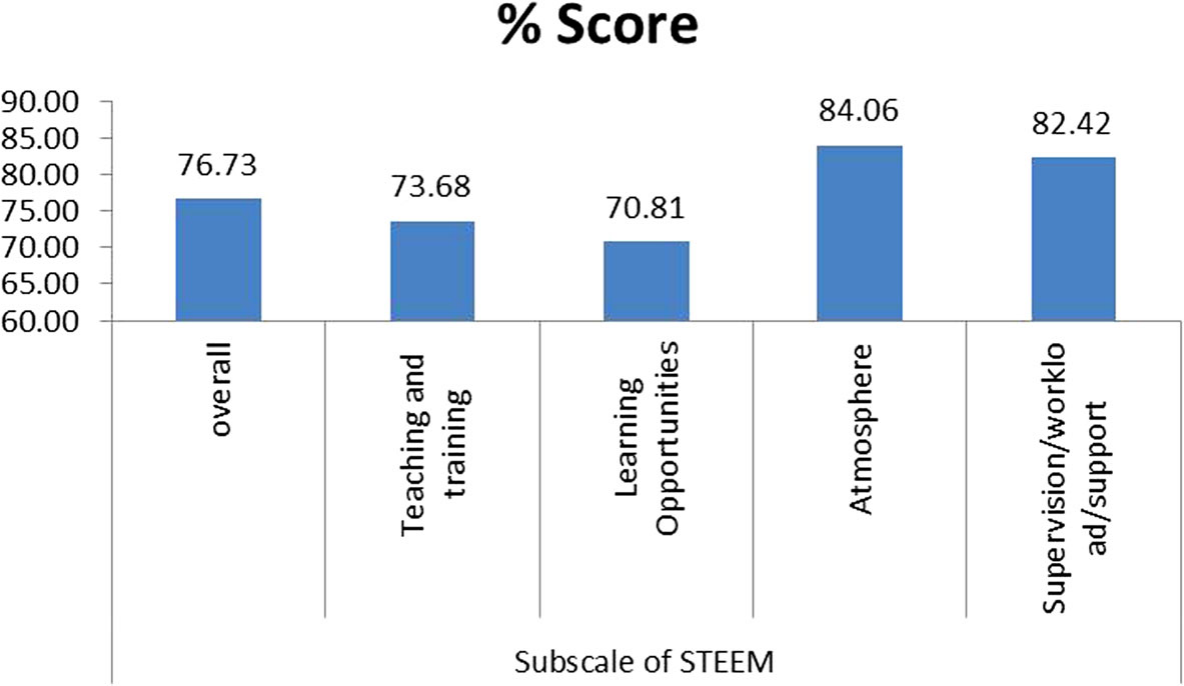
Overall percentage score and percentage scores for individual subscales for the whole group (*N* = 16)

The scores of subscales are depicted in Fig. 2 which shows the percentage scores for the whole group and individual subscales. The highest percentage score was given to the Atmosphere subscale with 427 out of 508 meaning an 84% score. The lowest score was given to Learning Opportunities with 490 out 692 which means a 70.8% score.

The most highly rated statements by the whole group were no. 17 “I get enough opportunity to assist”, no. 31 “I feel discriminated against in theatre because of my race”, no. 30 “I feel discriminated against in theatre because of my sex” and no. 36 “I am asked to perform operations alone that I do not feel competent at”. The medians were 4, and IQR were 4–4 and 3.5–4 respectively.

The lowest rated statements were no. 11 “My trainer expects my surgical skills to be as good as his/hers”. No. 20 “The number of emergency procedures is sufficient for me to gain the right operative experience”, no.21 “The variety of emergency cases gives me the appropriate exposure” and no. 22 “My trainer is in too much of a rush during emergency cases to let me operate”. The medians were 2, and IQR were 1–3, 2–2.5, 2–3 and 2–3 respectively.

## Discussion

The overall score of the study 122/160 (76.73%) demonstrates an overall satisfaction of the respondents. Using Likert’s [13] scale a score over 60% indicates a satisfactory learning environment but would aim for a very satisfactory score which would be 80% or higher. It has been suggested by Binsaleh [12] that a score less than 80% represents a learning environment that is less than agreeable, as it corresponds to a score lying between that of uncertain (60%) and agree (80%) on Likert’s non parametric scale. The Likert scale itself has shown to be a robust tool for parametric data however the sensitivity of then analysing the outcome has proven to be more challenging [14].

Table 3 shows a comparison between the current study and selected studies from the literature review. All the data has been adjusted to a 0–4 Likert scale to enable a true comparison which negates the overestimation of a 1–5 scale as recommended by Dimoliatis [11]. The current study does compare favourably to these studies which all have an overall score of less than 70% whereas the podiatric surgery learning environment shows a 76.73% agreement of a satisfactory environment. Cassar [10] in the original STEEM study suggests that his overall score of 67.9% showed a ‘satisfactory’ learning environment, however no reference was given as to how they came to deciding it was satisfactory. Therefore we can suggest that the current study demonstrates a satisfactory learning environment across the UK within podiatric surgery.

**Table 3:** Comparison between the current study and selected studies from the literature review

Variable	Cassar (2004)	Kanashiro (2006)	Mahony (2010)	Binsaleh (2015)	Current study
sample size	25	22	356	33	16
Response rate	25/26 (96%)	22/23 (95.6%)	356/1500 (24%)	33/72 (45.8%)	16/33 (48.4%)
Overall STEEM mean score	108/160	107/160	107.6/160	95.9/160	122/160
STEEM % score	67.90%	67.00%	67.30%	59.93%	76.73%
Subscale % Score					
Teaching and training	73.60%	66.30%	65.00%	58.84%	73.68%
Learning opportunities	59.40%	65.70%	65.00%	57.95%	70.81%
Atmosphere	70%	72.30%	72.50%	67.81%	84.06%
Supervision/workload/support	68.80%	59.40%	68.80%	56.56%	82.42%
	Vascular Surgery	General Surgery	All specialities	Urology	Podiatric Surgery

Subscales showed the Learning Opportunities as being the least satisfied for podiatric surgical trainees at 70.81%. However this appears to be a relatively good score compared to the other studies notably Cassar [10] and Binsaleh [12] being below 60%. There is a trend however across all the studies that this subscale is the one that is scored lowest. It is interesting to note the highest scored question is Q 17 “I get enough opportunity to assist” however the three of the lowest scored questions, Q 20, Q 21 and Q 22 “The number of emergency procedures is sufficient for me to gain the right operative experience”, “The variety of emergency cases gives me the appropriate exposure” and “My trainer is in too much of a rush during emergency cases to let me operate” contribute to the low Learning opportunities score as they have been scored poorly. It can be suggested these questions are inappropriate for podiatric surgery as the typical caseload is elective day case surgery [15]. It would seem appropriate to consider the meaning of emergency procedures within podiatric surgery and possibly remove these questions for future studies however the tool would require re-validation.

The strongest subscale was shown to be the ‘Atmosphere’ subscale and this is reflected across most of the previous studies. The Cassar [10] study however showed the ‘Teaching and Training’ to be the strongest. The strongest individual statements within the current study reflect this with Q 31 “I feel discriminated against in theatre because of my race” and Q 30 “I feel discriminated against in theatre because of my sex” highlighting the non-discriminatory atmosphere felt by the participants within the surgical theatre.

This study demonstrates that discrimination is scored very low. However Fnais et al. [16] in 2011 did a thorough systemic review and meta-analysis of discrimination and harassment within the medical trainee population showing it was still “surprisingly high”. This did suggest that the ‘consultant’ was the most commonly cited source for harassment and discrimination however this did include patient harassment towards the medical trainee.

The current study tries to reflect the podiatric surgery community within the UK and is potentially limited by the small sample size. In comparing the other studies that have utilised the STEEM there is a ‘like for like’ process. However it should be noted the working environment as well as the training within podiatric surgery is different to that of the medical specialities. Podiatric surgery units can operate within an acute setting, community setting or a mixture of the two and this would be of further interest in the future.

Either at a national or individual level, the studies can be utilised to assess the current situation of the learning environment, reflect what current students perceptions are and be used to compare to each other. This does potentially reflect each speciality and gives a comparison as to whether each speciality is giving a better or worse learning environment for the future of their profession. It may be that the current study has an advantage being contemporary as learning from previous studies and teaching and learning theories should hopefully advance over time giving better outcomes.

The original Cassar [10] study being 12 years ago will have set useful data for future studies to be able to compare to. It has been considered that participants may have some bias reporting on their own centre. However this bias has been mitigated for in utilising an anonymous survey.

## Conclusions

Perception was of a very satisfactory ‘Atmosphere’ within the theatre environment and a very satisfactory ‘opportunity to assist’ within the podiatric surgery Theatre environment. The results from this study can potentially be utilised within the monitoring and evaluation of the podiatric surgical theatre, to demonstrate a positive and supportive learning environment and potentially enhance the knowledge of the strengths and weaknesses within the learning environment.

This study provides clear evidence that the surgical trainee’s perception is one of a positive learning environment which includes being safe and supportive.

## Abbreviations

CCPST: Certificate of Completion of Podiatric Surgery Training
HCPC: Health and Care Professions Council
IQR: Interquartile Range
STEEM: Surgical Theatre Educational Environment Measure
